# From Syncope to Pregnancy: A Case Report of Gestational Transient Thyrotoxicosis

**DOI:** 10.7759/cureus.69739

**Published:** 2024-09-19

**Authors:** Ana Martins da Costa, Tiago Monteiro-Brás, Márcia Cravo, Guilherme Assunção

**Affiliations:** 1 Department of Internal Medicine, Centro Hospitalar Universitário de Santo António, Unidade Local de Saúde de Santo António, Porto, PRT; 2 Department of Hematology, Centro Hospitalar Universitário de Santo António, Unidade Local de Saúde de Santo António, Porto, PRT; 3 Department of Endocrinology, Diabetes, and Metabolism, Centro Hospitalar Universitário de Santo António, Unidade Local de Saúde de Santo António, Porto, PRT

**Keywords:** case report, gestational transient thyrotoxicosis, hcg, hyperemesis gravidarum, hyperthyroidism, pregnancy, syncope

## Abstract

With a multifactorial etiology, syncope is a fairly common clinical presentation in emergency care. During pregnancy, it can result from hemodynamic and hormonal changes. One such rare cause is hyperthyroidism. Although physiological maternal adaptation to fetal requirements can often induce mild, transient, and self-limited thyroid stimulation during the late first trimester, clinically significant thyrotoxicosis can still occur throughout pregnancy. In these cases, the most prevalent causes are gestational transient thyrotoxicosis (GTT) and Graves’ disease.

We report the case of a 24-year-old woman presenting to the emergency department with recurrent transient syncope, gastrointestinal symptoms, tachycardia, eyelid retraction, and tremor in the upper limbs. Laboratory analysis revealed electrolyte imbalances (metabolic alkalosis, hypokalemia, and hyponatremia), hepatic and kidney dysfunction, and a suppressed thyroid-stimulating hormone with a free thyroxine level of >7.77 ng/dL (reference range: 0.94-1.52 ng/dL), consistent with overt hyperthyroidism. After ruling out the most likely etiologies for syncope and thyroid dysfunction, including autoimmune causes, an abdominal ultrasound revealed the patient was 12 weeks pregnant. Extremely elevated serum human chorionic gonadotropin levels reinforced the presumed diagnosis of a GTT associated with hyperemesis gravidarum (HP). The patient started on fluid support with electrolyte replacement. Due to the uncommon clinical presentation, an antithyroid drug and a beta-blocker were also initiated. The patient showed clinical and biochemical improvement and was discharged after four days, with a close follow-up appointment scheduled with both the endocrinology and obstetrics outpatient care departments.

This case report highlights the necessity of considering hyperthyroidism as a potential etiology in cases of recurrent syncope, especially when clinical signs and symptoms are suspicious of excessive thyroid stimulation, while also reminding clinicians to consider pregnancy as a potential trigger in women of childbearing age.

## Introduction

Syncope occurs due to cerebral hypoperfusion, usually with an acute onset and a short and spontaneous recovery. Its main pathophysiologic classes are divided into reflex, cardiac, or orthostatic hypotension according to its etiology [1]. During pregnancy, these events occur due to hormonal and hemodynamic changes, the most common being vasovagal, with an estimated prevalence of 1% [2].

Although rare, clinical hyperthyroidism can occur in pregnancy in approximately 0.1% to 0.4% of gestations [3,4]. The most common cause of hyperthyroidism in pregnancy requiring treatment is Graves’ disease, estimated to account for 85% to 95% of clinically significant cases; a less common cause is gestational transient thyrotoxicosis (GTT), which is usually transient and does not require treatment [4-7]. GTT is exclusive to the first trimester, and its onset is directly related to human chorionic gonadotropin (hCG) serum levels [5]. hCG has a structural homology with the thyroid-stimulating hormone (TSH), allowing it to interact with thyroid TSH receptors and stimulate the production of thyroxine (T4) and, although in smaller quantities, of triiodothyronine (T3). As a result, TSH secretion decreases through negative feedback in the pituitary gland [8]. This physiological adaptation to serum hCG is usually mild and self-limited, peaking at the end of the first trimester with substantial improvement in the following trimester [9]. Due to its limited thyrotropic activity, considerable elevated concentrations of serum hCG are required to induce a clinically marked thyrotoxicosis [10]. Hyperemesis gravidarum (HG) is often associated with GTT due to their shared pathophysiology [3,9], occurring with a prevalence of 0.3% to 1% in pregnancies [10], characterized by a clinical presentation of nausea, vomiting, dehydration, and electrolyte depletion [8,10]. Severe forms of HG can increase the risk for maternal and fetal complications, with a need for close follow-up [10].

We present a case of thyrotoxicosis with atypical manifestations with a subsequent discovery of a pregnancy, posing a difficult challenge in the initial medical approach, differential diagnosis, and clinical management of this patient.

## Case presentation

A 24-year-old woman, without any significant medical, obstetric, or family history, presents to the emergency department with complaints of recurrent syncope with short and spontaneous recovery for the previous four days, occurring with minimal physical exertion. She also reported persistent fatigue, muscular weakness, perception of weight loss, anorexia, nausea, and several daily episodes of vomiting in the last two weeks. She denied experiencing heat intolerance, hyperdefecation, shortness of breath, or any other clinical symptom. She denied taking any medication or supplements.

**Figure 1:**
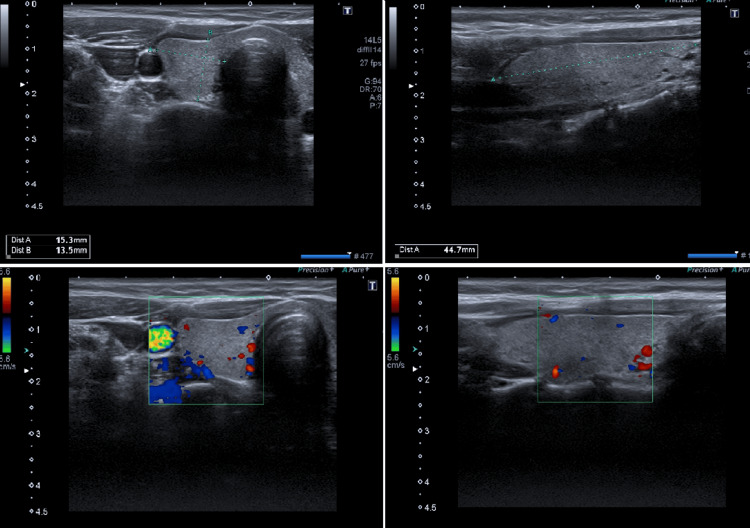
Thyroid ultrasound: thyroid gland with a normal size, homogeneous hyperechoic structure, no apparent nodules, and no increased vascularization on Doppler imaging

Upon physical examination, she was conscious and fully oriented, showing signs of dehydration such as xerostomia, reduced skin turgor, and tremor in the extremities of her upper body. She also presented with a fixed stare, lid lag, and mild eyelid retraction with no observable goiter. She had a normal body temperature, high arterial blood pressure (143/96 mmHg), and resting tachycardia (133 bpm). There were no other relevant signs on physical examination. An electrocardiogram revealed sinus tachycardia with no other abnormality. Laboratory analysis revealed hypochromic and microcytic anemia with a hemoglobin of 10.8 g/dL (reference range (RR): 12.0-15.0), a mean corpuscular volume of 72.3 fL (RR: 83.0-101.0) and a mean corpuscular hemoglobin of 25.8 pg (RR: 27.0-32.0), metabolic alkalosis with a pH of 7.603 (RR: 7.350-7.450), a partial pressure of oxygen of 99.0 mmHg (RR 83.0-108.0), a partial pressure of carbon dioxide of 32.8 mmHg (RR: 32.0-48.0) and a bicarbonate of 32.4 mmoL/L (RR: 22.0-26.0), hypokalemia of 2.2 mmoL/L (RR: 3.5-5.0), hyponatremia of 123 mmoL/L (RR: 135-148), abnormal liver chemistries with an aspartate transaminase of 225 U/L (RR: 10-30), an alanine transaminase of 358U/L (RR: 10-36); gamma-glutamyl transferase of 310 U/L (RR: 6-39); alkaline phosphatase of 205 U/L (RR: 35-104), acute kidney injury stage 1 with a creatinine of 1.56 mg/dL (RR: 0.50-0.90) and severe hyperthyroidism with a suppressed TSH (0.01 μIU/mL) and a free thyroxine (FT4) level of >7.77 ng/dL with no other abnormal parameters (Table 1). Parenteral fluid support was promptly initiated for hypovolemia along with electrolyte replacement and antiemetics. The Burch-Wartofsky Point Scale score indicated an impending thyroid storm (30 points for gastrointestinal-hepatic dysfunction and a heart rate of 133 bpm). Etiologies such as infection or trauma were excluded, along with thyroid auto-immune conditions, by measuring anti-TSH receptor antibody (TRAb), thyroid-stimulating immunoglobulin antibody (TSI), thyroid peroxidase (TPOAb), and anti-thyroglobulin (TgAb) antibodies, the results of which were all negative. A thyroid ultrasound was performed, revealing a thyroid gland with a normal size, homogeneous hyperechoic structure, no apparent nodules, and no increased vascularization on Doppler imaging (Figure 1). Due to the alterations in liver enzymes, an abdominal ultrasound was also performed, revealing a uterus with a single fetus (later determined to be at 12 weeks of gestation) without any other relevant change.

**Table 1:** Laboratory results upon arrival at the ED and at discharge TSH: thyroid-stimulating hormone; T4: thyroxine; T3: triiodothyronine

Parameters (units)	Values at admission	Values previous to discharge	Reference values
Hemoglobin (g/dL)	10.8	10.9	12.0-15.0
Mean corpuscular volume (fL)	72.3	74.8	83.0-101.0
Mean corpuscular hemoglobin (pg)	25.8	25.7	27.0-32.0
Creatinine (mg/dL)	1.56	0.54	0.50-0.90
Urea (mg/dL)	138	16	10-50
Total bilirubin (mg/dL)	1.95	0.55	0.20-1.00
Aspartate transaminase (U/L)	225	90	10-30
Alanine transaminase (U/L)	358	124	10-36
Gamma-glutamyl transferase (U/L)	310	99	6-39
Alkaline phosphatase (U/L)	205	106	35-104
Potassium (mmol/L)	2.20	4.05	3.50-5.00
Sodium (mmol/L)	123	135	135-148
TSH (µIU/mL)	0.01	0.01	0.33-4.59
Free T4 (ng/dL)	>7.77	2.63	0.94-1.52
Total T4 (μg/dL)	17.4	12.7	7.3-14.8
Free T3 (pg/mL)	5.02	3.91	2.46-3.89
Total T3 (ng/mL)	-----	1.60	1.05-2.30

After a multidisciplinary discussion, the patient was admitted for hemodynamic monitoring and started therapy with 100 mg of propylthiouracil (PTU) every six hours and 10 mg of propranolol every eight hours. Serum β-hCG levels were 198079,0 U/L, which suggested the diagnosis of GTT and HG. The patient exhibited notable progress, with gradual normalization of thyroid, renal, and liver function, allowing for the gradual tapering of PTU and propranolol, with withdrawal occurring after three days.

On discharge, the patient was asymptomatic, with no relevant findings on physical examination. Close follow-up appointments were scheduled, one post-admission consultation and another one with the endocrinology and obstetrics outpatient care departments, to which the patient did not comply.

## Discussion

Syncope is a common clinical presentation in emergency care [11]. It has several underlying mechanisms, such as impaired intravascular volume, abnormal cardiac output, and diminished systemic vascular resistance, which accounts for most of the syncope cases [1]. Thyroid conditions, namely hyperthyroidism, are a possible cause since decreased peripheral vascular resistance and increased ionotropic and chronotropic states can occur due to the stimulatory effect of excessive thyroid hormones [12]. During pregnancy, there are several mechanisms that might trigger syncope, such as hemodynamic changes, hormonal influences, vasovagal mechanisms, cardiac factors, orthostatic hypotension, dehydration, and anemia. The increased blood volume in pregnancy, due to increased metabolic demands, is accompanied by an augmented heart output. To accommodate such processes, vasodilation ensues, leading to sudden drops of blood pressure when moving from supine to seated or seated to orthostatic. Furthermore, the physiological adaptations, namely the increased serum progesterone levels, add another layer of vasodilation, leaving pregnant women especially susceptible to syncope. The higher cardiac output might also unmask previously undiagnosed structural heart conditions or lead to supraventricular tachycardias. Last, dilutional anemia or impaired volume due to dehydration can also be factors at play when dealing with syncope in pregnancy [2]. Healthy individuals can compensate for the cardiovascular effects of hyperthyroidism by increasing their cardiac output even further [13], but the synergic effect of hyperthyroidism and pregnancy leads to even lower vascular resistance and lower preload levels that might precipitate syncope [2,13].

During our initial approach and differential diagnosis, reminding that at this point we did not know the patient's pregnancy status, clinical findings such as fatigue, muscular weakness, perception of weight loss, tremors in the extremities of her upper body, as well as fixed stare with lid lag and mild eyelid retraction suggested GD. This auto-immune disorder is the most predominant cause of hyperthyroidism in iodine-sufficient regions and is characterized by the presence of stimulating TSHRAb. It affects young and middle-aged women four times more than men, and about 50% of these patients have a known family history of auto-immune disease [14]. As a young woman, the patient did fit in this group but had no personal or family history of auto-immune disease. She also presented with ocular manifestations that could suggest even more the possibility of GD, but these manifestations can happen in any form of thyrotoxicosis, as these appear to be caused by excessive adrenergic tone. Nevertheless, it is always important in an initial approach to a suspicion of GD to identify features suggestive of infiltrative orbitopathy [14]. Also, although there were characteristic clinical findings, the free T4 value was not in favor of GD. This condition usually presents with high serum T3 levels, sometimes with an increased FT3/FT4 ratio up to 20:1 [15]. The almost near-certain exclusion of a GD came when TSHRAb and TSI were revealed to be negative.

The pattern of serum thyroid hormone levels with a high ratio of T4/T3 is more common in the first phase of some forms of destructive thyrotoxicosis, such as autoimmune thyroiditis (post-partum or painless thyroiditis) or subacute thyroiditis [9]. Our patient did not present with an enlarged and/or painful goiter; infection was ruled out, just as a history of recent childbirth, and as mentioned above, she had no personal and familial history of thyroid or other auto-immune diseases. Also, TPOAb and TgAb were negative. Being rare conditions and even more scarce in pregnancy, the probability of a thyroiditis diagnosis was very low [9].

Regarding our patient's thyroid ultrasound, it did not reveal a heterogeneous pattern with increased vascularization or a diffuse thyroid enlargement that could be in favor of GD or thyroiditis [10].

After discovering the pregnancy, a serum β-hCG level was performed, showing an increased value (198079,0 U/L). By integrating all the clinical spectrum, it was presumed that the patient had thyrotoxicosis due to GTT in association with a severe HG.

One of the most prevalent forms of overt hyperthyroidism in pregnancy is GTT, which usually manifests as a moderate and limited increase in FT4 level [9]. It is a non-autoimmune form of excessive TSH receptor stimulation due to the elevation of serum hCG levels that predominantly occurs in the first trimester of pregnancy [10]. Its affinity to the TSH receptors promotes thyroid hormone production, although with limited thyrotrophic function, reaching its peak between the 10th and 12th weeks of gestation [15]. An extremely elevated hCG can be responsible for the development of severe thyrotoxicosis and severe HG, and in some cases, one or both of these identities can be associated with multiple gestations and trophoblastic tumors such as molar pregnancy or choriocarcinoma [9].

HG is a poorly defined medical condition that is diagnosed in the absence of clinical findings that make other conditions more likely. The most commonly cited criteria include persistent vomiting not related to other causes, a measure of acute starvation (usually large ketonuria), and some discrete measure of weight loss, most often at least 5% of pregnancy weight [16].

Regarding classical clinical manifestations of thyrotoxicosis, they may be similar in GTT and GD, but GTT is regularly associated with HG, and very often hyperemesis, anorexia, and dehydration are present. Analytical findings of GTT normally present overt hyperthyroidism with mildly elevated free T4 levels with an elevated T4/T3 ratio and low or suppressed TSH [6].

Symptom management is the cornerstone of GTT and/or HG treatment. Its focus is on controlling vomiting with antiemetics, managing fluid replacement, or if necessary, hospitalization [10]. If severe tachycardia is present, betablocking agents might be of use, for a limited time, due to fetal injury risk [8-10]. Anti-thyroid drugs (ATD) are not recommended due to the self-limited nature of this condition, gradually resolving and disappearing after the hCG peak [3,9,10,17]. After assessment of teratogenic and hepatotoxicity risk and due to the uncommon clinical presentation, the extremely elevated value of free T4, along with manifestations of overt hyperthyroidism, it was decided to start PTU on our patient. This ATD has less described teratogenic manifestations in the first semester of gestation when compared to methimazole but has a higher frequency of hepatotoxicity [4,17]. The risk of thyrotoxicosis complications varies depending on the severity and how long they last, but there are many described: gestational hypertension and pre-eclampsia, anemia, congestive heart failure, thyroid storm, low birth weight, early miscarriage, premature birth, even fetal death, but also cognitive, behavioral, and developmental changes during infancy have been described [9]. The notable clinical and biochemical improvement of the patient after the initiation of treatment strengthens the presumptive diagnosis of GTT and HG, allowing for a titration, and eventual withdrawal, of ATDs and beta-blockers within a short time. At discharge, the patient was clinically stable, and, although multiple early follow-up consultations were scheduled for close outpatient monitoring, the patient failed to attend, preventing us from knowing the final outcome of the patient.

Overall, GTT is frequently mild if overt hyperthyroidism is established, resolving almost spontaneously when the second semester starts, and is not usually associated with adverse maternal or fetal outcomes. If HG is present, supportive therapy management should be provided [17].

## Conclusions

This case highlights the importance of considering hyperthyroidism in the evaluation of recurrent syncope with clinical manifestations suggestive of excessive thyroid stimulation while also demonstrating the importance of including pregnancy in the differential diagnosis of thyrotoxicosis as a potential trigger in women of childbearing age.

GTT is often associated with HG and is usually self-limited and mild, although distinguishing between GGT and Graves’ disease in the first semester of gestation could prove to be challenging due to the possible overlap in clinical presentations. If isolated HG is suspected, thyroid function should be measured to rule out GTT, and if overt hyperthyroidism is confirmed, proper symptom management should be ensured. Although most women with GTT and HG will only need supportive therapy, correct management is essential to optimal maternal and fetal outcomes; therefore, in conditions with severe clinical manifestations of thyrotoxicosis, the use of ATDs should not be ruled out.
